# Renal Medullary Cancer in a Patient with Sickle Cell Trait

**DOI:** 10.1155/2013/129813

**Published:** 2013-10-07

**Authors:** Narendrakumar Alappan, Creticus P. Marak, Amit Chopra, Parijat S. Joy, Olena Dorokhova, Achuta K. Guddati

**Affiliations:** ^1^Division of Pulmonary and Critical Care Medicine, Montefiore Hospital, Albert Einstein College of Medicine, Yeshiva University, New York, NY, USA; ^2^Department of Internal Medicine, University of Iowa Hospital, University of Iowa, Iowa, IA, USA; ^3^Department of Pathology, Montefiore Hospital, Albert Einstein College of Medicine, Yeshiva University, New York, NY, USA; ^4^Department of Internal Medicine, Massachusetts General Hospital, Harvard Medical School, Harvard University, 50 Fruit Street, Boston, MA, USA

## Abstract

Renal medullary cancer is a rare malignancy almost exclusively seen in young patients of African ethnicity. These patients often present with the cardinal symptoms of hematuria, flank pain, and an abdominal mass, and this malignancy has been associated with patients carrying sickle cell trait. It is estimated that 300 million people worldwide carry sickle cell trait, and the presence of hematuria in these patients should be treated as a harbinger of a possible malignancy. Notably, this tumor mostly develops on the right side of the body. Patients often present with it at an advanced stage and the prognosis is poor. Therefore, a high index of suspicion in a patient of African descent presenting with a right sided abdominal mass and hematuria may assist in an early diagnosis. Current chemotherapy options are very limited, and early detection may provide a chance for surgical resection. It may also provide a bigger time frame for the initiation of novel chemotherapy regimens in patients who fail current chemotherapy regimens.

## 1. Introduction

Renal medullary cancer was described in 1995 by Davis et al. in a case series of 34 patients collected over 22 years. He reported a highly aggressive neoplasm with microscopic morphology of sickled erythrocytes in the tissue. Sickle cell trait was linked to all the cases except one patient who had hemoglobin SC disease [1]. To date, approximately 120 cases have been described in the medical literature, with only one report of its association with sickle cell disease [2]. Most patients present with the triad commonly seen in renal carcinoma, that is, gross hematuria, flank pain, and a palpable abdominal mass. Nearly 75% of the tumor masses are reported to be on the right side [3, 4]. Evidence of metastatic disease at the time of presentation is not uncommon. CT scan and MRI are imaging modalities of choice as there are instances of failure to detect a distinct renal mass using ultrasound [4]. Radiologically, the tumor tends to be poorly circumscribed, hypodense, and markedly infiltrative often with areas of internal necrosis, hemorrhage, and heterogeneity. Regional lymphadenopathy is common at presentation, as is distant metastatic disease to liver, lung, pleura, or omentum [5]. The relative low prevalence of this malignancy in patients with sickle cell disease is notable. It is possible that the sickle cell disease itself exerts a protective effect due to sickling of cells in the tumor vasculature and auto-infarcting it before the tumor grows further. However, there are no epidemiological studies to support this hypothesis. Here, we report the presentation and clinical course of a patient with sickle cell trait who presented with hematuria and was found to have renal medullary cancer.

## 2. Case Description

**Figure 1:**
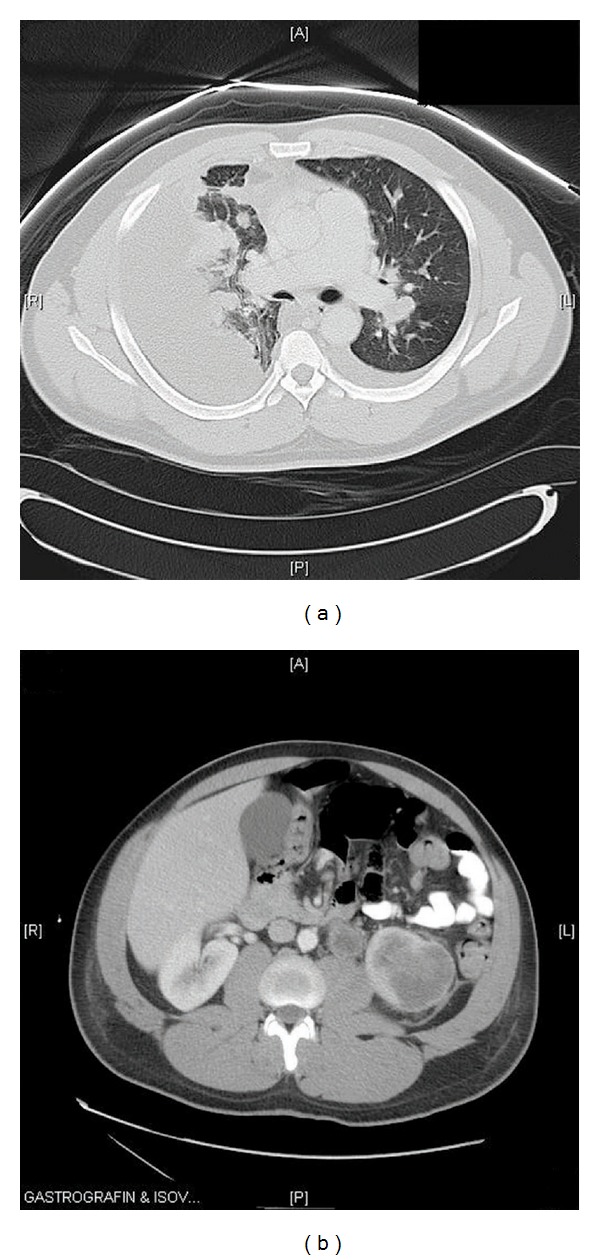
(a) CT scan of the chest in lung windows shows large right pleural effusion, small left pleural effusion, and multiple pulmonary nodules and masses. (b) CT scan of the abdomen shows irregular left lower pole low attenuation cystic renal mass with adjacent lymphadenopathy.

**Figure 2:**
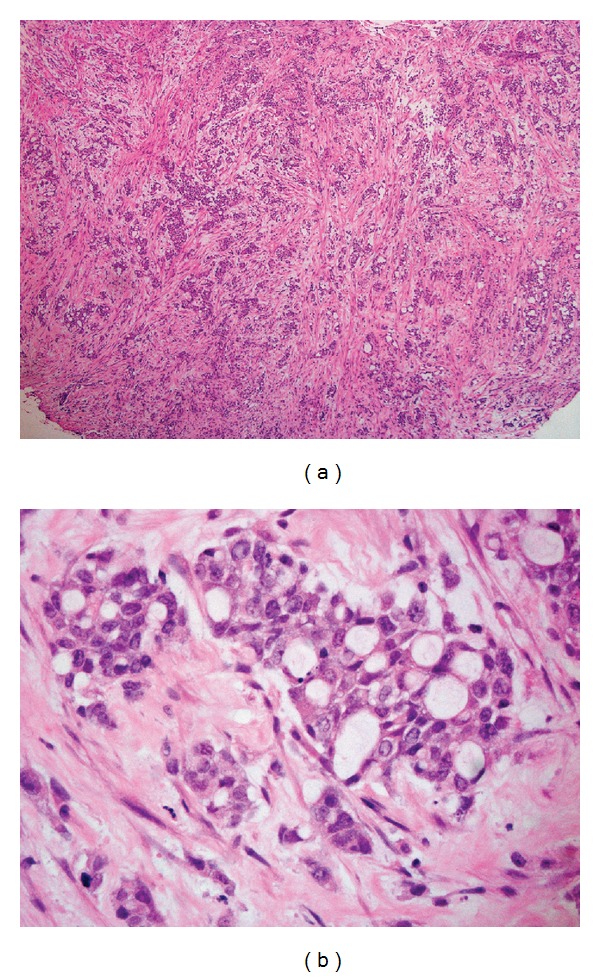
(a) Hematoxylin and eosin (H&E) staining showing reticular, solid, microcystic, adenoid cystic patterns, and desmoplastic stroma. (b) H&E staining showing pleomorphic cells with large vesicular nuclei with variable cytoplasm.

**Figure 3:**
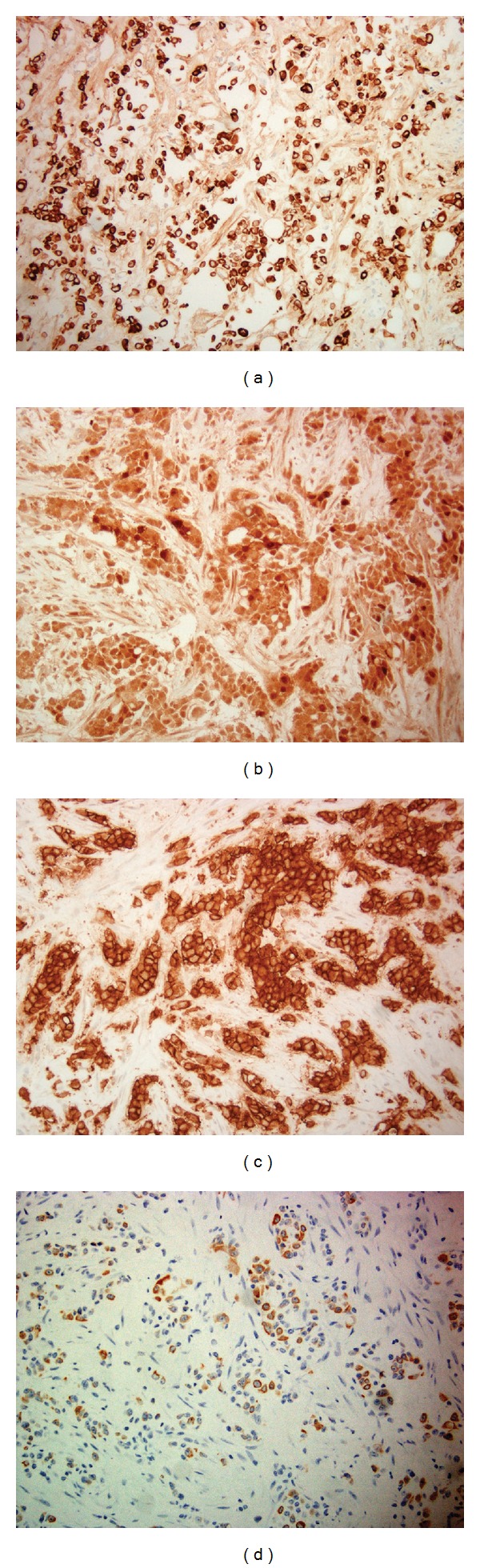
((a), (b), (c), and (d)) Immunohistochemistry showing positive staining for CK 7, calretinin, MOC31, and CK 20 (focal).

A 33-year-old African American gentleman, who immigrated to the United States from Cameroon 8 years ago presented with complaints of exertional shortness of breath, orthopnea, and nonproductive cough for duration of 1 week. He had a history of hypertension well controlled with amlodipine. He had been a life-long nonsmoker and denied use of any illicit drugs. He also denied any fever or chills or upper respiratory symptoms, oliguria or pedal edema. Review of other systems was unremarkable. On examination, he was noted to be in mild respiratory distress but was able to converse in full sentences. He was afebrile; blood pressure was 133/83 mmHg and heart rate was 91/minute, breathing at a rate of 24/minute with an oxygen saturation of 96% on ambient air. He did not have palpable cervical, axillary, or epitrochlear lymph nodes. His chest auscultation was significant for decreased breath sounds in the right base with dullness to percussion, and the left lung had clear vesicular breath sounds. His abdomen was nondistended, without any organomegaly. Initial routine laboratory investigation was significant for a normal white count with differentials, normal hemoglobin, and platelets, blood urea nitrogen of 11 mg/dL, and serum creatinine of 1.2 mg/dL. The urine analysis was remarkable for occult blood but had no protein or glucose. Chest X-ray (CXR) revealed a large right pleural effusion, a small left pleural effusion, and multifocal bilateral pulmonary opacities. This prompted a computed tomography (CT) scan of the chest and abdomen, which confirmed a large loculated right pleural effusion, small layering left pleural effusion, and multiple pleural based nodular enhancements, the largest of which measured 9.5 × 1.6 cm. There were innumerable pulmonary nodules throughout the left lung and visualized portion of the right lung. The left hilar lymph nodes were significantly enlarged. There was also a 5.8 × 5.2 × 5 cm irregular left lower pole low attenuation cystic renal mass, with adjacent 2.6 × 2.1 cm left para-aortic lymphadenopathy and 1 × 1 cm exophytic cyst from the lower pole of the right kidney (Figures 1(a) and 1(b)). The ultrasound of the scrotum revealed a 3 × 2.2 × 2.9 cm fluid collection superior to the right testis suggestive of spermatocele. Serum tumor markers like Alpha Feto-Protein and serum human Chorionic Gonadotropin were unremarkable. A therapeutic thoracentesis on the right chest was done, and 1.8 L of serosanguinous fluid was removed. Pleural fluid analysis revealed a white blood cell count of 1175/mm^3^, predominantly polymorphic neutrophils (70%), with lactate dehydrogenase of 271 U/L, glucose of 63 mg/dL and total protein of 5 g/dL, suggestive of an exudate. Cultures for bacterial, fungal, and acid fast bacilli were negative. Cytology was reported to be negative for malignant cells. Video-assisted thoracoscopic surgery (VATS) was requested to obtain definitive diagnosis. Inspection of the pleural cavity after evacuation of 1 L of bloody fluid during VATS revealed multiple deposits in both the parietal and visceral pleura, which were biopsied. Histopathological examination of the pleural biopsies revealed infiltrating tumor growth demonstrating reticular, solid, microcystic, adenoid cystic patterns and desmoplastic stroma. The cells were pleomorphic with large vesicular nuclei with variable cytoplasm (Figures 2(a) and 2(b)). Immunohistochemistry was positive for cytokeratins: CK7, CK19, CK20 (focal staining), CK903, epithelial glycoprotein-2 (MOC31), epithelial cell adhesion molecule (BerEp), epithelial membrane antigen (EMA), mucin 1 (MUC1), vimentin, and calretinin (Figures 3(a), 3(b), 3(c), and 3(d)). They were negative for thyroid transcription factor-1 (TTF1), napsin; caudal type homeobox transcription factor 2 (CDX2), carcinoembryonic antigen (CEA); and human epidermal growth factor receptor 2 (Her2/neu), O-linked sialoglycoprotein (MW 40 kDa) [D2-40], transformation-related protein 63 (p63), cytokeratins 5/6 (CK5/6), placental alkaline phosphatase (PLAP), AFP, CD30, and alpha-methylacyl CoA racemase (p504S). These features were indicative of poorly differentiated adenocarcinoma of an unknown primary. Given the African American ethnicity of the patient, renal mass and metastatic poorly differentiated adenocarcinoma, a diagnosis of metastatic renal medullary cancer was considered. This diagnosis was further supported when hemoglobin electrophoresis results were found to have a sickle cell trait (HbS-40.4% and HbA-55.6%). His post-operative period was otherwise uneventful. Unfortunately, he was lost to further follow up.

## 3. Discussion

Sickle cell trait (HbAS) is characterized by the inheritance of an abnormal sickle cell B1-globin gene along with normal hemoglobin gene (HbA) resulting in alpha2/betaS1beta1 hemoglobin configuration [6]. Largely considered as a benign carrier state usually with none of the symptoms of sickle cell disease, it offers relative protection against severe malaria. Current cumulative evidence is convincing for associations with hematuria, renal papillary necrosis, hyposthenuria, splenic infarction, exertional rhabdomyolysis, and exercise-related sudden death [7]. The prevalence of sickle cell trait is approximately 8–10 percent in African Americans, and it is as high as 25–30 percent in certain areas of western Africa [8, 9]. Individuals with sickle cell trait do not appear to have increased mortality rate as compared to the general population.

Compared to all the benign manifestations of sickle cell trait described above, renal medullary carcinoma is a rare and aggressive tumor that is seen almost exclusively in young patients with sickle cell trait [1]. The tumor is hypothesized to arise from the epithelium of the distal collecting ducts and grows in an infiltrative pattern, invading the renal sinuses. The genetic changes related to these tumor types are still largely unknown. Previous reports of immunohistochemical studies suggested the association of expressions of tumor protein 53 (TP53), vascular endothelial growth factor (VEGF), or hypoxia inducible factor (HIF) with the tumorigenesis [10]. Increased amplification of Abelson gene and absence of SWI/SNF-related matrix-associated actin-dependent regulator of chromatin subfamily B member 1 protein may also be involved in the pathogenesis of renal medullary carcinoma [11, 12]. In gross appearance, the tumor tends to be lobulated, firm, and poorly circumscribed. On histopathological examination, it reveals a cohesive group of cells with vacuolated cytoplasm, displaced or indented nuclei, and prominent nucleoli. Both the Davis et al. and Swartz et al. series demonstrated reticular pattern, cytoplasmic inclusions resembling rhabdoid tumor, and a high incidence of tumor hemorrhage [1, 10]. 

Management options for renal medullary carcinoma include radical nephrectomy, chemotherapy, and palliative radiation therapy. The median survival is 15 months [7]. Simpson et al. and Swartz el al. reported the longest survivals for 46 weeks and 15 months, respectively [10, 11]. Motzer et al. reported a case of complete response to bortezomib at 27 months of follow up [13]. A recent report of rearrangements in anaplastic lymphoma kinase (ALK) receptor kinase as a possible mechanism for the tumorigenesis in this carcinoma opens the possibility of use of direct and indirect ALK inhibitors in the management of these patients [14]. Early detection may help decide the possibility of surgical resection and the initiation of chemotherapy regimens. Failure of these interventions may pave the path for possible participation in chemotherapy trials with novel agents and help enhance our understanding of the management of this rare malignancy.
